# The HeyL-Aromatase Axis Promotes Cancer Stem Cell Properties by Endogenous Estrogen-Induced Autophagy in Castration-Resistant Prostate Cancer

**DOI:** 10.3389/fonc.2021.787953

**Published:** 2022-01-12

**Authors:** Qimei Lin, Jiasong Cao, Xiaoling Du, Kuo Yang, Yongmei Shen, Weishu Wang, Helmut Klocker, Jiandang Shi, Ju Zhang

**Affiliations:** ^1^ Department of Biochemistry and Molecular Biology, College of Life Sciences, Bioactive Materials Key Laboratory of the Ministry of Education, Nankai University, Tianjin, China; ^2^ Tianjin Key Lab of Human Development and Reproductive Regulation, Tianjin Central Hospital of Obstetrics and Gynecology, Nankai University, Tianjin, China; ^3^ Department of Urology of the Second Hospital of Tianjin Medical University, Tianjin, China; ^4^ State Key Laboratory of Medicinal Chemical Biology and College of Life Sciences, Nankai University, Tianjin, China; ^5^ Department of Urology, Division of Experimental Urology, Medical University of Innsbruck, Innsbruck, Austria

**Keywords:** CRPC, HeyL, aromatase, autophagy, prostate cancer stem cell

## Abstract

Treatment of patients with castration-resistant prostate cancer (CRPC) remains a major clinical challenge. We previously showed that estrogenic effects contribute to CRPC progression and are primarily caused by the increased endogenous estradiol produced *via* highly expressed aromatase. However, the mechanism of aromatase upregulation and its role in CRPC are poorly described. In this study, we report that HeyL is aberrantly upregulated in CRPC tissues, and its expression is positively correlated with aromatase levels. HeyL overexpression increased endogenous estradiol levels and estrogen receptor-α (ERα) transcriptional activity by upregulating *CYP19A1* expression, which encodes aromatase, enhancing prostate cancer stem cell (PCSC) properties in PC3 cells. Mechanistically, HeyL bound to the *CYP19A1* promoter and activated its transcription. HeyL overexpression significantly promoted bicalutamide resistance in LNCaP cells, which was reversed by the aromatase inhibitor letrozole. In PC3 cells, the HeyL-aromatase axis promoted the PCSC phenotype by upregulating autophagy-related genes, while the autophagy inhibitor chloroquine (CQ) suppressed the aromatase-induced PCSC phenotype. The activated HeyL-aromatase axis promoted PCSC autophagy *via* ERα-mediated estrogenic effects. Taken together, our results indicated that the HeyL-aromatase axis could increase endogenous estradiol levels and activate ERα to suppress PCSC apoptosis by promoting autophagy, which enhances the understanding of how endogenous estrogenic effects influence CRPC development.

## Introduction

Prostate cancer (PCa) is the most common cancer and the second leading cause of cancer mortality in men (1). The current cornerstone treatment for locally advanced or metastatic PCa is androgen deprivation therapy (ADT), which includes reducing androgen production and inhibiting androgen receptor (AR) activity (2). Although the effect of ADT is temporary, it eventually leads to lethal castration-resistant prostate cancer (CRPC) (3). Based on the theory that the proliferation and survival of most PCa cells depend on the androgen signaling pathway, previous efforts have revealed the role of restored AR signaling (such as increased testosterone levels within the tumor), AR bypass signaling, and complete AR independence in CRPC (4–6). On the other side of the coin, the upregulated expression and activity of estrogen receptor-α (ERα) and ERα‐regulated genes (e.g., *MAPK*, *PI3K*, the *TMPRSS2‐ERG* fusion, and *NEAT1*) during the progression of PCa to CRPC suggests that tumors can bypass the AR signaling axis by using estrogens for their growth (7–9). Prostate cancer stem cells (PCSCs) are considered an essential cell subpopulation in CRPC (10). Our previous studies have already shown that PCSCs exhibit higher ERα levels and that estradiol enhances their basal cell-like phenotype *via* the ERα-NOTCH1 axis, thereby promoting epithelial-mesenchymal transition (11).

Previous studies have confirmed that human prostate stem cells, albeit AR negative or low expression of AR, express high levels of ERα and exhibit a proliferative response to estrogen (12, 13). Furthermore, brief estrogen exposure epigenetically reprograms prostate stem cells and predisposes them to estrogen-driven carcinogenesis (14). Accumulating mutations of prostate stem cells located in the basal layer of the prostate gland are considered to be one of the source of PCSCs (15), which plays an important role in cancer relapse and CRPC progression (16, 17). Aromatase, encoded by the *CYP19A1* gene, is a key enzyme that catalyzes the formation of estrone and estradiol from testosterone and androstenedione (18). In mice with CYP19A1 knockout, the absence of estrogen production was found to prevent PCa development despite the increased testosterone levels (19). Aromatase can also promote metastatic homing and the growth of PCSCs in the bone marrow (20). Our recent research found that aromatase expression was significantly increased in CRPC tissues and that bicalutamide treatment upregulated *CYP19A1* expression in PCa cell lines. An increased level of aromatase-generated endogenous estradiol could drive metastasis through ERα/MMP12 axis activation in the CD44^+^ subpopulation of PC3 cells (21). However, the molecular mechanism underlying aromatase expression in CRPC and the role and mechanism of aromatase-induced estrogenic effects in bicalutamide resistance remain in their infancy.

HeyL is an essential downstream effector of the Notch pathway and is highly expressed in some estrogen-related tumors (22). Elevated HeyL levels have been discovered in breast cancer, and HeyL transgenic mice display accelerated mammary gland epithelial proliferation, eventually leading to breast cancer (23). Activation of the Notch signaling pathway has been reported to enhance the activity of ALDH1A1^+^ breast cancer stem cells and promote tamoxifen resistance (24). In contrast, inhibiting the Notch signaling pathway was found to reduce the estradiol levels during the development of ovine ovarian follicular granulosa cells (25), suggesting that the Notch signaling pathway may play a critical role in regulating estrogenic effects. However, in PCa and CRPC, the function of HeyL remains to be revealed.

In the present study, we explored the underlying regulatory mechanism linking HeyL and aromatase in CRPC and investigated the role and molecular mechanism of aromatase-induced endogenous estradiol in bicalutamide resistance and maintenance of PCSC properties.

## Materials and Methods

### Cell Culture and Reagents

PCa cell lines (LNCaP, PC3, and 22Rv1) were obtained from the American Type Culture Collection (ATCC, Manassas, VA, USA), and LNCaP-abl cells were a gift from Professor Helmut Klocker (Innsbruck University School of Medicine) (26). LNCaP, PC3, and 22Rv1 cells were cultured in RPMI 1640 medium (Gibco, Grand Island, NY, USA) containing 10% fetal bovine serum (FBS, Gibco, Grand Island, NY, USA), and LNCaP-abl cells were cultured in RPMI 1640 medium supplemented with 10% charcoal:dextran stripped fetal bovine serum (CS-FBS, Invitrogen, Carlsbad, CA, USA). Bicalutamide (Selleck Chemicals, Houston, TX, USA) was used at a concentration of 20 μM. Letrozole (MCE, Monmouth Junction, NJ, USA) was used at 50 nM. Chloroquine (CQ; Solarbio, Beijing, China) was used at 20 μM. All cells were incubated at 37°C in a humidified atmosphere containing 5% CO_2_.

For the tumorsphere formation assay, LNCaP-abl cells (2000 cells) were suspended in DMEM/F12 medium containing 1 μL/mL transferrin (Sigma-Aldrich, Saint Louis, Missour, USA), 20 ng/mL EGF (Peprotech, Rocky Hill, NJ, USA), 20 ng/mL basic FGF (Peprotech, Rocky Hill, NJ, USA), 2% B27 (Invitrogen, Carlsbad, CA, USA) and 10 units/mL human LIF (Sigma-Aldrich, Saint Louis, Missour, USA) and were then seeded in ultra-low attachment 6-well plates (Corning, Amsterdam, The Netherlands). Tumorspheres were visualized under a light microscope and counted after two weeks of culture in a humidified incubator at 37°C containing 5% CO_2_.

### RNA Extraction and Quantitative Real-time Polymerase Chain Reaction (qRT-PCR)

Total RNA was extracted with the TRIzol reagent (Invitrogen, Carlsbad, CA, USA) according to the manufacturer’s instructions. Complementary DNA (cDNA) was synthesized with a HiScript 1st Strand cDNA Synthesis Kit (Vazyme, Nanjing, China). After the qRT-PCR assay, the relative transcription levels of the indicated genes were determined by normalization to the transcript level of the housekeeping gene *HPRT* and calculation by the 2^−ΔΔCt^ method. A single-cell sequence-specific amplification kit (Vazyme, Nanjing, China) was used to analyze gene expression in tumorspheres that contained only a few cells. The sequences of the primers used here are shown in **Supplementary Table 1**.

### Western Blot Analysis

Total protein was extracted by using RIPA buffer (Beyotime, Shanghai, China). The protein concentration was determined with a BCA protein assay kit (Beyotime, Shanghai, China). 40μg of denatured protein were separated by sodium dodecyl sulfate-polyacrylamide gel electrophoresis (SDS-PAGE) and transferred to a polyvinylidene fluoride (PVDF) membrane (Millipore, Bedford, MA, USA). After blocking with 5% nonfat dry milk in TBST, the membrane was incubated with primary antibodies and horseradish peroxidase-conjugated secondary antibodies. Bands were detected using an ECL Western Blotting Substrate (Thermo Fisher; Waltham, USA). The primary antibodies used here were as follows: rabbit polyclonal anti-HeyL (ab26138, Abcam, Cambridge, UK, 1:1000), rabbit polyclonal anti-CYP19A1 (HPA051194, Sigma-Aldrich, Saint Louis, Missour, USA, 1:1000), rabbit polyclonal anti-CD44 (15675-1-AP, Proteintech, Chicago, USA, 1:2000), rabbit polyclonal anti-SOX2 (ab92494, Abcam, Cambridge, UK, 1:1000), mouse monoclonal anti-tubulin (KM9003, SUNGENE Biotech, Tianjin, China, 1:5000), rabbit polyclonal anti-Beclin1 (ab62557, Abcam, Cambridge, UK, 1:1000), and rabbit polyclonal anti-LC3B (NB600-1384, NOVUS Biologicals, Minneapolis, MN, USA, 1:1000).

### Colony Formation and Cell Viability Assays

For the colony formation assay, 1000 cells were seeded in 6-well plates and treated with vehicle (DMSO), bicalutamide or letrozole. Colonies were counted after two weeks of culture in a humidified incubator at 37°C containing 5% CO_2_.

For the evaluation of cell viability, 5000 cells were seeded into a 96-well plate and treated with 20 μM bicalutamide for 48 h. Cell viability was evaluated with an MTT Cell Growth Assay Kit (Sigma-Aldrich, Saint Louis, Missour, USA).

### Immunohistochemical (IHC) and Immunofluorescence (IF) Analyses

Antibodies against HeyL (F7241-1F6, Sigma-Aldrich, Saint Louis, Missour, USA, 1:500) and CYP19A1 (HPA051194, Sigma-Aldrich, Saint Louis, Missour, USA, 1:200) were used to validate protein expression in 9 benign prostate tissues, 23 primary PCa tissues and 15 CRPC tissues. All clinical samples were obtained with the informed consent of the patients, and the study was approved by the Ethics Committee of Nankai University. IHC and IF analyses were performed on prostate tissue as described previously (27). IHC staining images were acquired with an Olympus BX43 microscope (Tokyo, Japan), and the optical density of the image was analyzed using the ImageJ software. IF staining images were acquired with a Zeiss LSM719 confocal microscope (Germany).

### Plasmid Construction and Lentiviral Transduction

The coding sequence of HeyL was synthesized and subcloned into pcDNA3.1(+), and the *CYP19A1*-pENTER plasmid was purchased from ViGene Biosciences Inc. The plasmids were transfected into PCa cells as described previously (27). The CRISPRi sgRNA sequence targeting the human HeyL gene was subcloned into the letiSAM2-dCas9-KRAB vector to obtain CRISPRi_HeyL. A sgRNA oligonucleotide that did not match any known human DNA sequence was used as a control. CRISPRi lentiviral particles were packaged in HEK293T cells and transduced into PC3 or LNCaP-abl cells. Stable cell lines were obtained after selection with 10 μg/mL blasticidin (Solarbio, Beijing, China).

The efficiency of overexpression or knockdown was determined by qRT-PCR and Western blot analysis. The sequences of the primers used here are shown in **Table S2**.

### Chromatin Immunoprecipitation (ChIP) and Luciferase Reporter Assays

The anti-HeyL antibody (F7241-1F6, Sigma-Aldrich, Saint Louis, Missour, USA) was used to capture HeyL-binding chromatin segments using an EZ-Magna ChIPA/G Chromatin Immunoprecipitation Kit (Merck Millipore, Darmstadt, Germany) in strict accordance with the manufacturer’s specifications. Primers complementary to the promoter region of *CYP19A1* (forward: 5’-cacaaaatgactccacctctgg-3’; reverse: 5’-caagtcaaaacaaggaagcc-3’) were used to detect *CYP19A1* genomic DNA. For the luciferase reporter assay, pGL3-AROM-promoter-luciferase reporter plasmids and pTK-RL were cotransfected into PC3 cells, and luciferase activity was evaluated with a Dual-Luciferase Assay Kit (Promega, Madison, WI, USA).

### Flow Cytometry Analyses

For analysis of CD44^+^/CD24^-^ PCSCs, PCa cells were dissociated into single cells, resuspended in HBSS buffer containing 10% BSA, and incubated with anti-CD44-APC (Affymetrix eBioscience, ThermoFisher Scientific, Waltham, MA, USA) and anti-CD24-PE (Affymetrix eBioscience, ThermoFisher Scientific, Waltham, MA, USA) antibodies for 15 min on ice. The CD44^+^ and CD24^-^ subpopulations were analyzed using flow cytometry (BD Biosciences, San Diego, CA, USA).

For the apoptosis assay, cells were exposed to DMSO or to bicalutamide and/or letrozole for 24 h. After washing with PBS buffer, cells were collected and incubated with FITC-conjugated Annexin V and propidium iodide (PI) according to the protocol of the Cell Apoptosis Detection Kit (US Everbright Inc., Suzhou, China). Apoptosis was analyzed by flow cytometry.

### Bioinformatic Analysis

*PCa patient data* The Cancer Genome Atlas (TCGA) prostate adenocarcinoma cohort dataset, which contains data for 498 PCa patients, was obtained from the Genomic Data Commons (GDC) data portal (https://portal.gdc.cancer.gov/). Heatmap analysis and hierarchical clustering based on the highest and lowest HeyL expression values (top and bottom quartiles from the TCGA dataset) were generated using Morpheus (https://software.broadinstitute.org/morpheus). The gene set enrichment analysis (GSEA) software package (GSEA3.0.jar) and molecular markers provided by the BroadIn Research Institute were used for GSEA. Survival and correlation analyses were performed using GEPIA2 (http://gepia2.cancer-pku.cn).

*RNA sequencing (RNA-Seq) and analysis* Total RNA was extracted using the TRIzol Reagent (Invitrogen, Carlsbad, CA, USA) according to the manufacturer’s protocol and then sequenced on the Illumina HiSeq 4000 platform. Reads were mapped to the reference genome (GRCh37/hg19). Quantitative analysis of the transcript abundance of each gene was performed through the HTSeq-DESeq2 pipeline, and saw in **Table S3**.

### Statistical Analysis

All statistical analyses were performed with Prism 8.0 software (GraphPad Software Inc., USA). The data are presented as the means ± SD of three independent experiments. The Student’s t-test was performed to analyze differences between the two groups, and ANOVA was performed to analyze differences among more than two groups. A p value < 0.05 was considered to be statistically significant.

## Results

### HeyL Overexpression Is Associated With Enhanced Estrogenic Effects in CRPC

**Figure 1:**
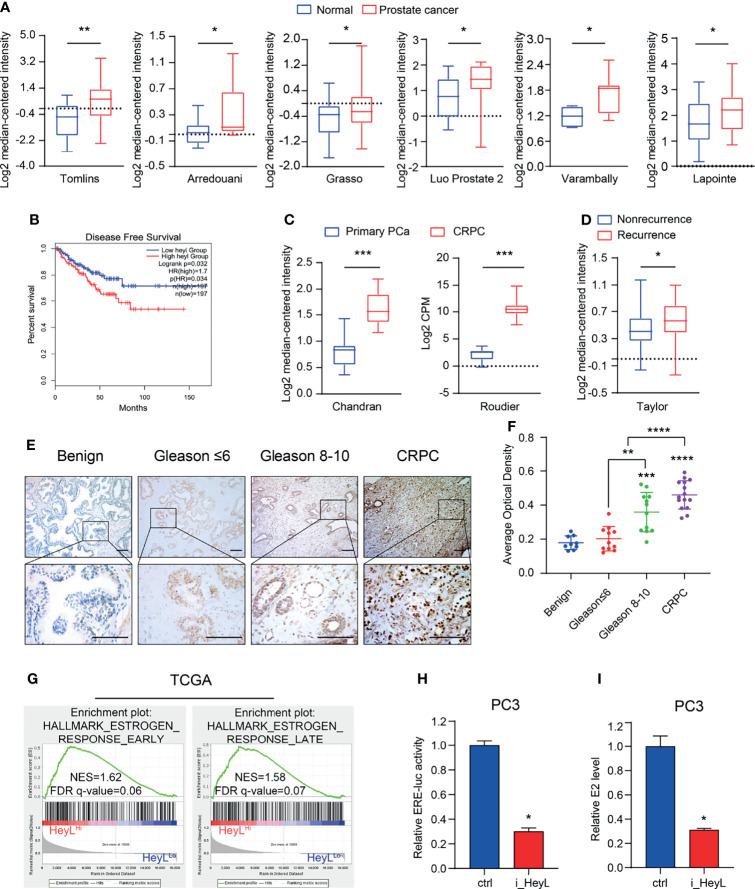
HeyL overexpression is associated with poor prognosis and estrogenic effects in patients with CRPC. **(A)** *HeyL* mRNA levels in normal prostate and PCa tissues from 6 publicly available datasets (Tomlins, n(normal)=23, n(PCa=29); Arredouani, n(normal)=8, n(PCa=13); Grasso, n(normal)=28, n(PCa=59); Luo Prostate 2, n(normal)=15, n(PCa=15); Varambally, n(normal)=6, n(PCa=7); Lapointe, n(normal)=40, n(PCa=60)) (t-test). **(B)** Kaplan-Meier curve of disease-free survival differences according to the *HeyL* expression level based on a TCGA cohort. **(C)** *HeyL* mRNA expression in primary PCa and CRPC tissues from 2 publicly available datasets (Chandran, n(primary PCa)=10, n(CRPC=21); Roudier, n(primary PCa)=11, n(CRPC=45)) (t-test). **(D)** Comparison of *HeyL* mRNA expression between recurrent and nonrecurrent PCa (n(nonrecurrent)=104), n(recurrent)=36) (t-test). **(E)** Representative images of HeyL IHC staining in benign prostate (n=9), low Gleason score PCa (Gleason score ≤ 6, n=11), high Gleason score PCa (Gleason score 8-10, n=12), and CRPC tissues (n=15); scale bar: 100 µm. **(F)** Average optical density of HeyL in low Gleason score vs. high Gleason score tissues and in primary PCa (low Gleason score and high Gleason score) vs. CRPC tissues (t-test). **(G)** GSEA plot of the association between HeyL and the estrogen response gene set in the TCGA-PRAD dataset. **(H, I)** ER transcriptional activity (ERE-Luc) **(H)** and level of intracellular 17β-estradiol **(I)** in control and HeyL knockdown PC3 cells. PRAD: prostate adenocarcinoma. The data are presented as the mean ± SD values (n=3). *p < 0.05, **p < 0.01, ***p < 0.001, ****p < 0.0001.

We examined the expression of Notch signaling downstream effectors in the TCGA and Oncomine databases and found that *HeyL* expression was significantly higher in PCa than in normal tissues (**Figure 1A** and **Figures S2, S3**). Increased *HeyL* expression may lead to a lower disease-free survival (DFS) rate among PCa patients (**Figure 1B**). *HeyL* expression was significantly upregulated in CRPC tissues compared to primary PCa tissues (**Figure 1C**), and *HeyL* expression was higher in the relapsed patient subset than in the nonrelapsed patient subset (**Figure 1D**). IHC analysis was carried out to evaluate the protein expression of HeyL in benign prostate (n=9), primary PCa (n=23), and CRPC (n=15) samples. HeyL was expressed in some basal epithelial cells, especially in the nucleus, in benign prostate samples. In primary PCa samples, tumors with a high Gleason score (Gleason 8-10) had stronger HeyL staining than those with a low Gleason score (Gleason ≤ 6). In addition, the CRPC tissues exhibited higher HeyL expression than the primary PCa tissues (**Figures 1E, F**). These results highlight the clinical significance of HeyL in CRPC.

To determine the potential mechanism regulated by HeyL, we individually selected prostate adenocarcinoma samples in the top quartile of the HeyL^High^ and HeyL^Low^ groups from TCGA and performed GSEA on these samples. Signatures representative of early estrogen response and late estrogen response were enriched in the HeyL^High^ group (**Figure 1G**). In addition, the correlation of HeyL expression with that of estrogen response gene sets was higher in PCa than in normal prostate tissues (**Figure S4**). As expected, the ERE-Luc reporter assay showed that HeyL knockdown reduced the luciferase activity in PC3 cells (**Figure 1H** and **Figure S5**), and high-performance liquid chromatography-mass spectrometry (HPLC-MS) demonstrated that the concentration of intracellular 17β-estradiol decreased after HeyL knockdown (**Figure 1I**).

### HeyL Increases the Endogenous Estradiol Concentration by Directly Upregulating *CYP19A1* Expression in CRPC

**Figure 2:**
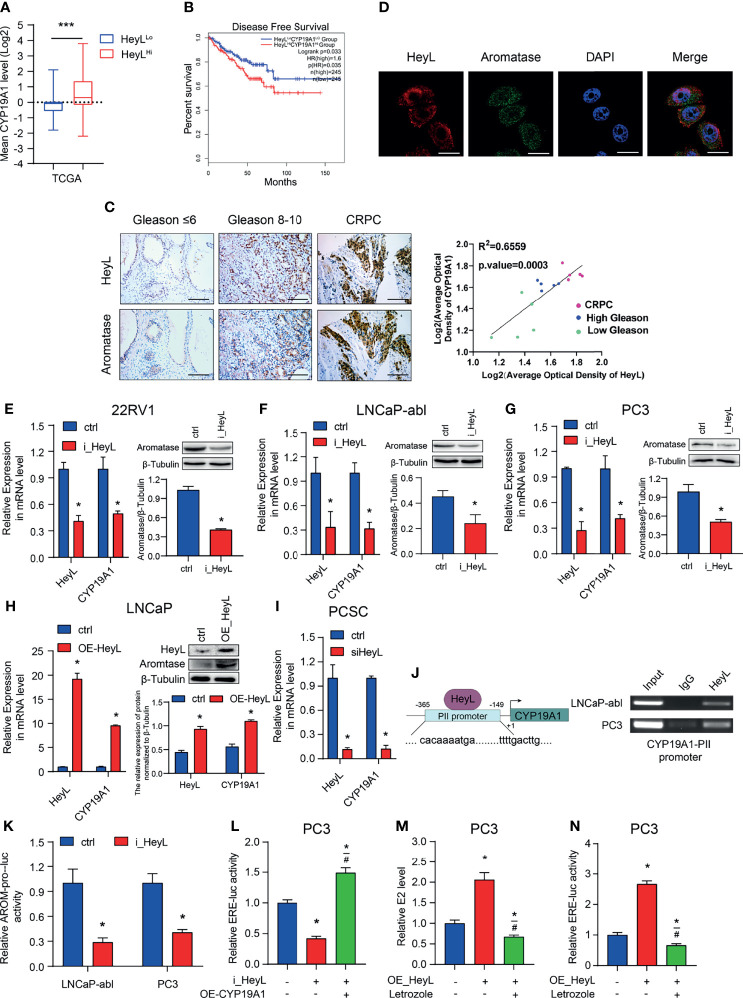
HeyL promotes endogenous estrogenic effects by directly activating *CYP19A1* transcription. **(A)** Mean mRNA expression levels of *CYP19A1* in HeyL^High^ samples vs. HeyL^Low^ samples in the TCGA-PRAD dataset. **(B)** Kaplan-Meier analysis of disease-free survival for HeyL^High^CYP19A1^High^ patients vs. HeyL^Low^CYP19A1^Low^ patients in the TCGA-PRAD dataset. **(C)** Representative images of HeyL and aromatase IHC staining in low Gleason score (Gleason score ≤ 6, n=5), high Gleason score PCa (Gleason score 8-10, n=5), and CRPC (n=5) tissues. Scale bar: 100 μm. The average optical densities of HeyL and aromatase were calculated, followed by correlation analysis of HeyL and aromatase expression in primary PCa and CRPC specimens. **(D)** Colocalization of HeyL and aromatase in PC3 cells was detected by IF staining. Scale bar: 20 μm. **(E–I)** Effects of HeyL on aromatase expression. **(E–G)** The mRNA (left panel) and protein (right panel) levels of aromatase in 22RV1, LNCaP-abl, and PC3 cells stably transfected with control or i_HeyL were analyzed (t-test). **(H)** The mRNA (left panel) and protein (right panel) levels of aromatase in ctrl or HeyL-overexpressing LNCaP cells were analyzed (t-test). **(I)** Relative mRNA level of aromatase in PCSCs transfected with siNC or siHeyL (t-test). **(J, K)** HeyL directly activates *CYP19A1* transcription by occupying its promoter. **(J)** Left: Schematic diagram of the binding site between HeyL and the promoter of *CYP19A1* promoter. Right: ChIP showed that HeyL binds directly to the *CYP19A1* PII promoter region. **(K)** Relative aromatase promoter activity (AROM-pro-Luc) in LNCaP-abl and PC3 cells stably transfected with control or i_HeyL (t-test). **(L–N)** Relative ERE-Luc activity **(L, N)** and the concentration of intracellular 17β-estradiol **(M)** in PC3 cells after the indicated treatment (one-way ANOVA). PCSC, prostate cancer stem cell; ChIP, chromatin immunoprecipitation; E2, 17β-estradiol. The data are presented as the mean ± SD values (n=3). *p < 0.05 vs. ctrl. ^#^ *p* < 0.05 vs. i_HeyL or OE-HeyL. ***p < 0.001.

Our previous study revealed that elevated aromatase expression promotes intracellular estrogenic effects in PCa, especially in CRPC (21). Here, we explored the relationship between HeyL and aromatase. The mean expression level of the *CYP19A1* gene was significantly higher in HeyL^High^ prostate adenocarcinoma samples than in HeyL^Low^ prostate adenocarcinoma samples (**Figure 2A**). In addition, PCa patients with high *CYP19A1*/*HeyL* levels had worse disease-free survival outcomes than those with low *CYP19A1*/*HeyL* levels (**Figure 2B**). IHC analysis showed that the expression of both aromatase and HeyL in CRPC was significantly higher than that in primary PCa tissues. Moreover, regardless of the primary PCa or CRPC, the expression of HeyL and aromatase showed a high degree of consistency in the tissue localization of serial sections (**Figure 2C** and **Figure S6**). Furthermore, HeyL and aromatase showed good coexpression in PC3 cells by confocal microscopy (**Figure 2D**).

Next, we analyzed the expression level of *CYP19A1* after interfering with the HeyL level in PCa cells. HeyL knockdown markedly decreased aromatase mRNA and protein expression in 22Rv1, PC3, and LNCaP-abl cells (**Figures 2E–G**), while HeyL overexpression in LNCaP cells increased aromatase levels (**Figure 2H**). A similar result was found in PCSCs enriched from LNCaP-abl cells (**Figure 2I**). Since the *CYP19A1* PII promoter was aberrantly activated in PCa cell lines, we designed primers targeting this promoter region to assess the HeyL binding sites. The ChIP assay revealed that HeyL was recruited to the *CYP19A1* PII promoter in PC3 and LNCaP-abl cells (**Figure 2J**), and the luciferase reporter assay showed that HeyL knockdown significantly decreased *CYP19A1* PII promoter activity (**Figure 2K**).

Knockdown of HeyL reduced aromatase expression and ERE promoter activity. We transfected the *CYP19A1* plasmid into HeyL-silenced PC3 cells and found that re-expression of aromatase rescued the luciferase activity of the ERE reporter and the concentration of endogenous estradiol (**Figures 2L, M**). In contrast, HeyL overexpression in PC3 cells significantly upregulated ERE promoter activity, while treatment with letrozole, an aromatase inhibitor, markedly inhibited ERE promoter activity (**Figure 2N**). These findings indicated that HeyL promotes endogenous estrogenic effects by increasing the aromatase level.

### The Activated HeyL-Aromatase Axis Is Essential for Promoting PCSC Properties

**Figure 3:**
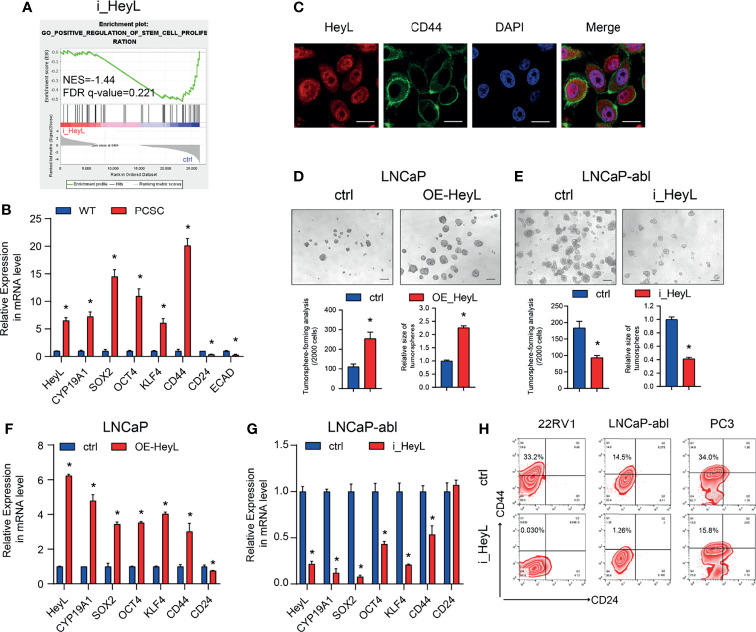
HeyL functions to maintain the characteristics of PCSCs. **(A)** GSEA plot of the correlation between *HeyL* expression and gene signatures associated with stem cell proliferation in HeyL knockdown cells. **(B)** Relative mRNA levels of *HeyL*, *CYP19A1*, *CD44*, and the indicated stemness-associated genes in PCSCs enriched from LNCaP-abl cells (t-test). **(C)** Colocalization of HeyL and CD44 in PC3 cells was detected by IF staining. Scale bar: 20 μm. **(D, E)** Tumorsphere-forming ability of LNCaP and LNCaP-abl cells treated as indicated. The top panel shows representative micrographs (scale bar: 100 μm); the bottom panel shows the quantitative results [**(D)**, LNCaP, t-test; **(E)**, LNCaP-abl, t-test). **(F, G)** The mRNA levels of *CD44* and the indicated stemness-associated genes in HeyL-overexpressing LNCaP cells **(F)** and HeyL knockdown LNCaP-abl cells **(G)** (t-test). **(H)** Flow cytometry analysis of the CD44^+^/CD24^-^ subpopulation in 22RV1, LNCaP-abl, and PC3 cells after HeyL knockdown. PCSC, prostate cancer stem cell. The data are presented as the mean ± SD values (n=3). *p < 0.05 vs. ctrl.

GSEA of HeyL knockdown cells and the corresponding control cells revealed that HeyL expression was positively correlated with signatures representative of stem cell proliferation (**Figure 3A**). The expression of *HeyL*, together with the stemness-associated genes *SOX2*, *OCT4*, *KLF4*, and *CD44*, was significantly increased in PCSCs compared with the corresponding bulk cancer cells (**Figure 3B**). Moreover, HeyL and CD44 were co-expressed in a subset of PC3 cells (**Figure 3C**). Correspondingly, HeyL overexpression in LNCaP cells increased the number and size of tumorsphere compared with control cells, while HeyL knockdown in LNCaP-abl cells decreased them (**Figures 3D, E**). In addition, overexpression of HeyL increased the mRNA levels of these stemness-associated genes in LNCaP cells, and knockdown of HeyL significantly decreased the expression of these genes in LNCaP-abl cells (**Figures 3F, G**). CD44^+^/CD24^-^ cells were defined as PCSCs (28). As expected, flow cytometry analysis showed that knockdown of HeyL significantly decreased the CD44^+^/CD24^-^ PCSC subpopulation (**Figure 3H**). Additionally, the expression of *CYP19A1* was significantly increased under sphere culture conditions compared with attachment culture conditions (**Figure 3B**), and knockdown of CYP19A1 markedly reduced the tumorsphere-forming capacity, decreased the CD44^+^/CD24^-^ PCSC subpopulation, and decreased the expression of stemness-associated genes (**Figure S7**).

**Figure 4:**
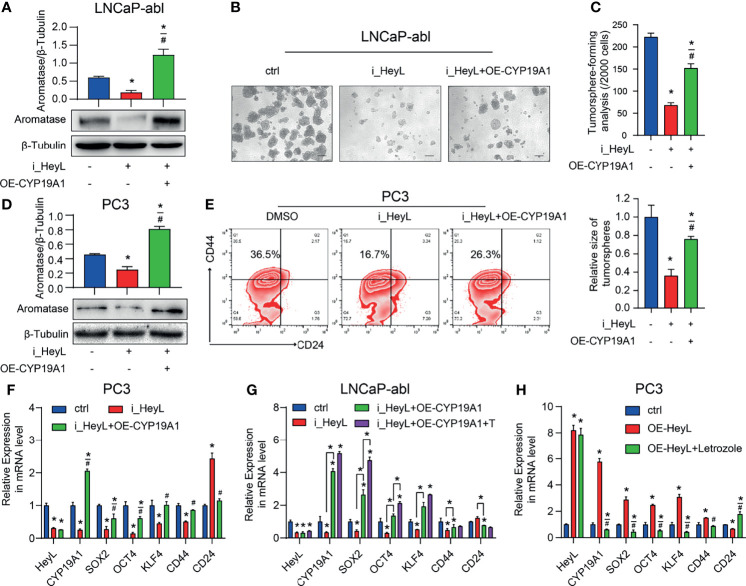
HeyL regulates PCSC properties by upregulating *CYP19A1* expression. **(A–C)** Tumorsphere-forming ability of LNCaP-abl cells treated as indicated. The protein levels of aromatase, representative micrographs, and quantitative results (one-way ANOVA) are shown in **(A)**, **(B)**, and **(C)**, respectively. Scale bar: 100 μm. **(D, E)** Flow cytometry analysis of the CD44^+^/CD24^−^ subpopulation in PC3 cells treated as indicated. **(D)** Protein levels of aromatase. **(E)** The results of flow cytometry analysis. **(F–H)** mRNA levels of *CD44* and the indicated stemness-associated genes in PC3 **(F, G)** and LNCaP-abl **(H)** cells treated as indicated (one-way ANOVA). The data are presented as the mean ± SD values (n=3). *p < 0.05 vs. ctrl. ^#^ *p* < 0.05 vs. i_HeyL or OE-HeyL.

To validate whether HeyL regulates PCSC properties by upregulating *CYP19A1* expression, we rescued aromatase expression by transfecting HeyL-silenced cells with *CYP19A1*-pENTER. Re-expression of CYP19A1 in HeyL-silenced PCa cells restored the tumorsphere-forming capacity (**Figures 4A–C**), the proportion of CD44^+^/CD24^-^ PCSCs (**Figures 4D, E**), and the expression of stemness-associated genes (**Figures 4F, G**). In addition, HeyL overexpression in PC3 cells significantly upregulated stemness-associated genes, but letrozole markedly suppressed this effect (**Figure 4H**). These results suggest that aromatase is essential for HeyL-mediated effects on PCSC properties.

### The Aromatase Inhibitor Letrozole Reverses HeyL-Induced Bicalutamide Resistance in LNCaP Cells

**Figure 5:**
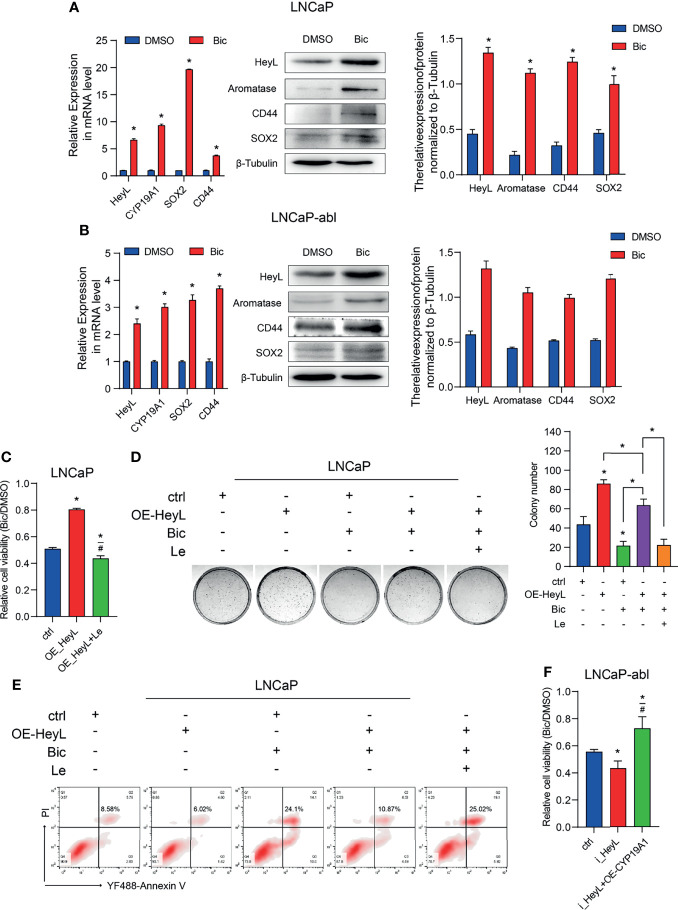
Blocking the HeyL-aromatase axis sensitizes PCa cells to bicalutamide treatment. **(A, B)** The mRNA (left panel) and protein (right panel) levels of HeyL, aromatase, SOX2, and CD44 were analyzed in LNCaP **(A)** and LNCaP-abl **(B)** cells treated with bicalutamide (t-test). **(C)** Cell viability was evaluated in LNCaP cells after the indicated treatments (one-way ANOVA). **(D)** The clonogenic ability of vector control and HeyL-overexpressing LNCaP cells treated with bicalutamide and/or letrozole was analyzed (one-way ANOVA). **(E)** Vector control LNCaP cells and HeyL-overexpressing LNCaP cells were treated with bicalutamide and/or letrozole. Apoptosis was evaluated by flow cytometry. **(F)** Cell viability was evaluated in LNCaP-abl cells after the indicated treatments (one-way ANOVA). The data are presented as the mean ± SD values (n=3). *p < 0.05 vs. ctrl. ^#^ *p* < 0.05 vs. OE-HeyL.

PCSCs play a crucial role in the recurrence of CRPC. We next determined whether the activated HeyL-aromatase axis enhances bicalutamide resistance in PCa cells. After treatment with bicalutamide for 1 week, the expression of HeyL and aromatase increased significantly in both LNCaP and LNCaP-abl cells, accompanied by increased expression of CD44 and SOX2 (**Figures 5A, B**). HeyL-overexpressing LNCaP cells exhibited increased survival and colony formation, accompanied by a reduction in apoptosis, while these effects were abolished after letrozole treatment (**Figures 5C–E**). However, HeyL knockdown markedly promoted the inhibitory effect of bicalutamide on cell viability, while exogenous CYP19A1 overexpression increased the viability of HeyL knockdown cells upon bicalutamide treatment (**Figure 5F**). These results further support the significant role of the HeyL-aromatase axis in the bicalutamide resistance of PCa cells.

### The Activated HeyL-Aromatase Axis Suppresses PCSC Apoptosis by Promoting Autophagy

**Figure 6:**
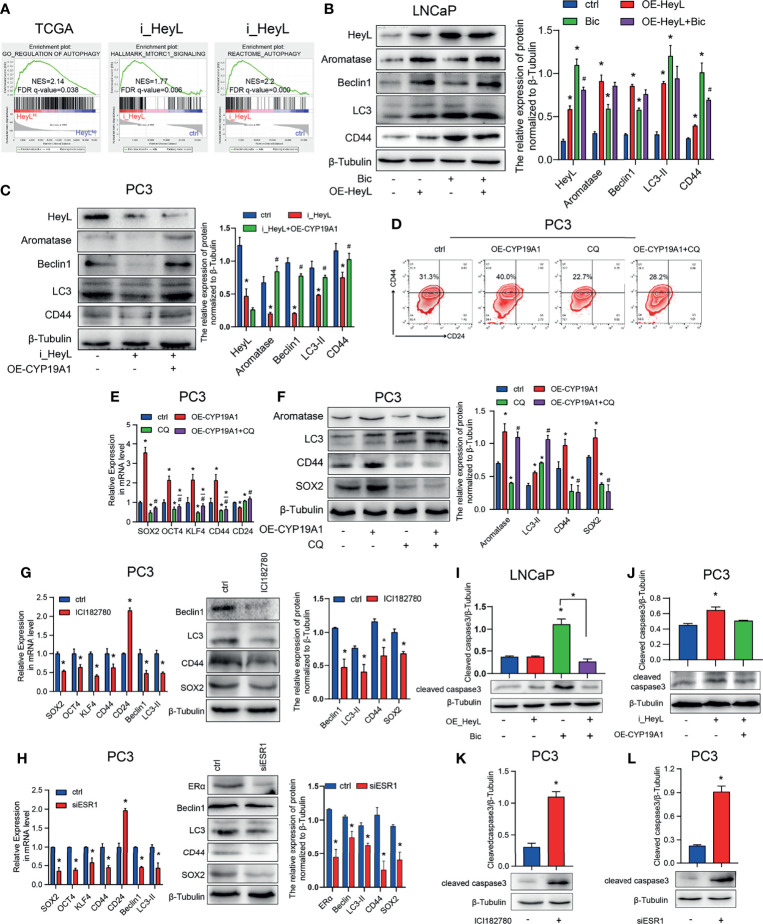
The activated HeyL-aromatase axis promotes autophagic survival of PCSCs. **(A)** Gene signatures associated with autophagy-associated signaling pathways identified by GSEA in HeyL^High^ samples vs. HeyL^Low^ samples from the TCGA-PRAD dataset and in HeyL knockdown cells vs. ctrl cells. **(B, C)** Western blot analysis of Beclin1, LC3, and CD44 in LNCaP **(B)** and PC3 **(C)** cells after the indicated treatments. **(D)** Flow cytometry analysis of the CD44^+^/CD24^−^ subpopulation in PC3 cells treated as indicated. **(E, F)** mRNA **(E)** and protein **(F)** levels of stemness-associated genes and LC3 in PC3 cells after the indicated treatment (one-way ANOVA). **(G, H)** mRNA and protein levels of stemness-associated genes and autophagy-related genes in PC3 cells treated with ICI182780 **(G)** and siERα **(H)** (t-test). **(I, J)** Cleaved caspase3 levels in LNCaP and PC3 cells after the indicated treatments (one-way ANOVA). **(K, L)** Cleaved caspase3 levels in PC3 cells treated with ICI182780 **(K)** or transfected with siERα **(L)** (t-test). Bic, bicalutamide; CQ, chloroquine. The data are presented as the mean ± SD values (n=3). *p < 0.05 vs. ctrl. ^#^ *p* < 0.05 vs. OE-HeyL, i_HeyL or OE-CYP19A1.

Autophagy contributes to CRPC progression (29). To investigate the underlying mechanism by which the HeyL-aromatase axis regulates bicalutamide sensitivity, we conducted GSEA with HeyL^High^ and HeyL^Low^ prostate adenocarcinoma samples from the TCGA dataset and found that HeyL expression was positively correlated with signatures representative of autophagy regulation (**Figure 6A**). Notably, in the RNA-Seq data from 22Rv1 cells, knockdown of HeyL was positively enriched among genes associated with MTORC1-related signatures and negative regulation of the autophagy (**Figure 6A**). LC3 and Beclin1 are known autophagy signaling molecules. In LNCaP cells, overexpression of HeyL dramatically increased the levels of LC3, Beclin1, and CD44 (**Figure 6B**). In addition, knockdown of HeyL in PC3 cells decreased the expression of LC3, Beclin1, and CD44, while overexpression of CYP19A1 in HeyL knockdown cells increased the levels of these proteins (**Figure 6C**). To explore the potential role of autophagy in regulating PCSC properties in CRPC cells, we treated CYP19A1-overexpressing PC3 cells with CQ, an inhibitor of autophagosome-lysosome fusion. As shown in **Figures 6D–F**, the promotive effects of aromatase on PCSC properties were decreased by CQ. In addition, blocking estrogen signaling with the ER inhibitor ICI182780 or knocking down ERα in PC3 cells decreased the expression of stemness-associated genes and autophagy-associated genes (**Figures 6G, H**).

To determine whether the autophagy pathway regulated by the HeyL-aromatase axis mediates the survival of PCSCs, we assessed apoptosis in PCa cells. In bicalutamide-treated LNCaP cells, HeyL overexpression increased CD44 expression and decreased the BAX/BCL2 ratio and cleaved caspase3 levels (**Figure 6I** and **Figures S8A, B**), while knockdown of HeyL in PC3 cells reduced the CD44 levels and increased the BAX/BCL2 ratio and cleaved caspase3 levels (**Figure 6J** and **Figures S8C, D**). PC3 cells treated with ICI182780 or ERα knockdown displayed an increased BAX/BCL2 ratio and cleaved caspase3 levels (**Figures 6K, L** and **Figures S8E, F**). Accordingly, we found that blocking ER signaling in HeyL-overexpressing LNCaP-abl cells attenuated the tumorsphere-forming capacity (**Figure S9**). Blocking ER signaling in HeyL-overexpressing PC3 cells reduced the expression of stemness-associated genes and autophagy-associated genes, while increased the BAX/BCL2 ratio (**Figure S10**). These results suggest that the activation of autophagy induced by the HeyL-aromatase axis is involved in inhibiting apoptosis in PCSCs.

## Discussion

Our series of studies shows that enhanced estrogenic effects play a critical role in promoting the progression of CRPC. Aromatase is highly expressed in CRPC tissues, and the elevated endogenous estradiol produced by this enzyme is the major cause of the enhanced estrogenic effect (21). However, the precise molecular mechanism by which *CYP19A1* expression is upregulated in CRPC remains unknown. HeyL has been reported to repress GATA-binding protein 4 (GATA4)-dependent *CYP19A1* promoter activation in small follicles (30). In addition, the Notch signaling pathway decreases the *CYP19A1* levels in mouse ovarian granulosa cells by inhibiting the expression of upstream transcription factors, such as steroidogenic factor 1 (SF1), Wilms’ tumour 1 (Wt1), Gata4 and Gata6 (31). Kaplan-Meier analysis of the TCGA prostate adenocarcinoma (TCGA-PRAD) dataset indicated that patients with combined high expression of *HeyL* and *CYP19A1* had lower disease-free survival rates than those with combined low expression of *HeyL* and *CYP19A1*. Moreover, the expression of both HeyL and aromatase was significantly increased in CRPC. In PCa cells, HeyL upregulated CYP19A1 expression, thus increasing the endogenous estradiol concentration and ERα transcription activity. A ChIP assay showed that HeyL can bind to the *CYP19A1* PII promoter, suggesting that the mechanism by which HeyL regulates *CYP19A1* expression exhibits apparent specificity between different pathological conditions and tissue types.

PCSCs are an important cause of ADT failure (32). In PCa, the subset of cells expressing CD44 but lacking CD24 (CD44^+^/CD24^-^) are characterized as PCSCs (33). In this study, we found that the levels of HeyL and CYP19A1 were increased in CD44^+^/CD24^-^ PCSCs. Knocking down either HeyL or CYP19A1 significantly reduced PCSC properties, including the proportion of CD44^+^/CD24^-^ PCSCs, tumorsphere formation ability, and the expression of stemness-associated genes. In contrast, overexpressing CYP19A1 in HeyL knockdown PC3 cells reversed these PCSC properties. Furthermore, the expression levels of HeyL, aromatase, and stemness-associated genes were significantly increased in LNCaP and LNCaP-abl cells treated with bicalutamide. Overexpression of HeyL in LNCaP cells significantly enhanced bicalutamide resistance, while blocking HeyL signaling with letrozole (an aromatase inhibitor) rescued the sensitivity of cells to bicalutamide, suggesting that the activated HeyL-aromatase axis in PCa cells can enhance endogenous estrogenic effects, thus attenuating the sensitivity of PCa cells to bicalutamide by promoting PCSC properties. It has been proposed that prostate cancer stem cells can be originated from prostate stem cells located in basal layer of the prostate gland which hardly express PSA (15, 34). Gallee et al. indicates that PSA^-/low^ cell subsets enriched and sorted from LNCaP cells have stronger clonogenic ability, tumorigenesis, and invasiveness than PSA^+^ cell subsets (35). We found that HeyL was expressed mainly in basal epithelial cells in benign prostate tissues and showed intense nuclear staining. In addition, we observed that overexpression of HeyL in LNCaP cells downregulated the expression of PSA, a classical AR downstream target gene, but had no effects on AR expression (**Figure S11**), consistent with the results of Derek et al. (36). These results indicated that HeyL may enhance PCSC properties in the ADT environment by upregulating CYP19A1 expression in CRPC and that HeyL may also maintain the poorly differentiated state of PCSCs by stabilizing low AR signaling.

**Figure 7:**
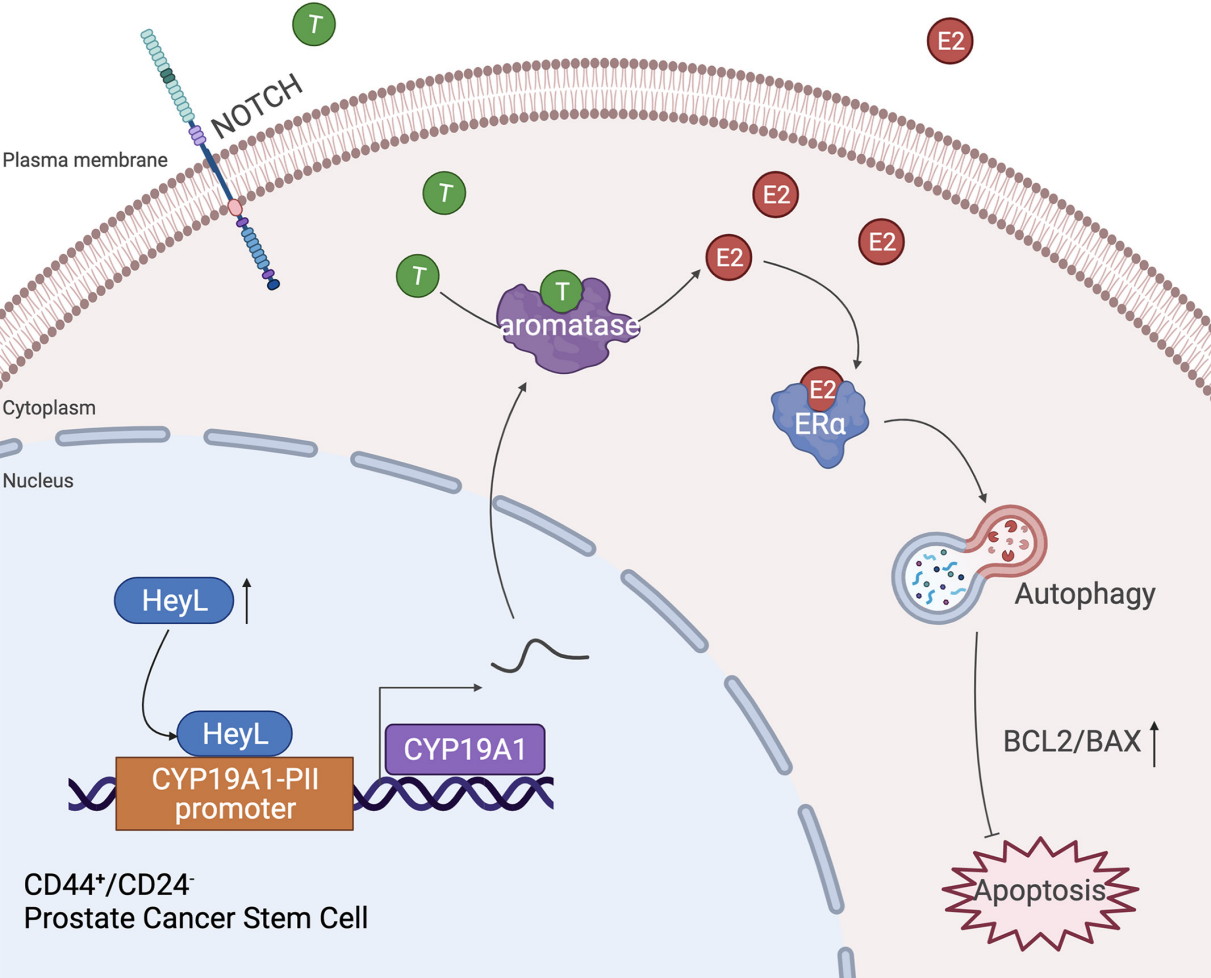
Schematic depiction of the proposed mechanism by which the HeyL-aromatase axis promotes stemness maintenance and apoptosis resistance in CD44^+^/CD24^-^ PCSCs. CRPC cells exhibit higher levels of HeyL and aromatase than androgen-dependent PCa cells. HeyL promotes *CYP19A1* expression by directly binding to the *CYP19A1* PII promoter region. The activated HeyL-aromatase axis enhances the stemness of the CD44^+^/CD24^-^ subpopulation and attenuates bicalutamide sensitivity. The residual testosterone derived from serum and intratumoral biosynthesis after castration is catalyzed to estradiol by the HeyL-aromatase axis in CRPC patients. Increased levels of intracellular estradiol enhance ERα-induced autophagy, thereby promoting the apoptotic resistance of the CD44^+^/CD24^-^ subpopulation. T, Testosterone; E2, Estrogen/Estradiol; ERα, Estrogen receptor α.

Autophagy is a conserved lysosome-associated pathway that maintains cellular homeostasis and promotes cell survival by degrading and recycling cellular components under certain stress conditions (37, 38). Bicalutamide treatment has been reported to induce autophagy in hormone-resistant PCa cells and confer resistance to apoptosis (39). Here, we found that the expression level of HeyL was positively correlated with the early and late estrogen response signaling pathways and the autophagy-regulated signaling pathway. Overexpression of HeyL in LNCaP cells upregulated the expression of autophagy-related genes and stemness-associated genes, and knockdown of HeyL in PC3 cells significantly downregulated the expression of those genes and attenuated the PCSC phenotypes. The effects of HeyL knockdown in PC3 cells were reversed by CYP19A1 expression. In addition, CQ, an inhibitor of autophagosome-lysosome fusion, significantly reversed the aromatase-induced upregulation of autophagy-related genes and stemness-associated genes and increased the proportion of PCSCs among PC3 cells. Furthermore, in PC3 cells, knockdown of ERα expression with small interfering RNA (siRNA) or treatment with the ER inhibitor ICI182780 reduced the expression levels of autophagy-related genes and attenuated the PCSC phenotype, accompanied by an increase in the BAX/BCL2 ratio and cleaved caspase 3 levels. These data suggest that the increased estrogenic effects induced by the activated HeyL-aromatase axis can suppress PCSC apoptosis by enhancing autophagy, which contributes to CRPC development (**Figure 7**). However, the precise molecular mechanism by which estradiol regulates autophagy in PCSCs needs to be elucidated in future experiments.

In conclusion, this study elucidated the molecular mechanism of the increased endogenous estrogenic effects in CRPC and the possible role and mechanism of these effects in promoting bicalutamide resistance in PCa cells.

## Data Availability Statement

The datasets presented in this study can be found in online repositories. The names of the repository/repositories and accession number(s) can be found in the article/**Supplementary Material**.

## Author Contributions

QL designed and conducted the study, analyzed the data and drafted the manuscript. JC contributed to flow cytometric analysis and bioinformatic analysis. XD performed pathological analysis. KY provided clinical specimens. YS and HK participated in data analysis. WW completed the plasmid construction and lentiviral transfection. JS and JZ provided study materials, helped with data analysis and drafted the manuscript. All authors read and approved the final manuscript.

## Funding

This work was supported by grants from the National Natural Science Foundation of China (No. 81872087, No. 82073119 and No. 81772687).

## Conflict of Interest

The authors declare that the research was conducted in the absence of any commercial or financial relationships that could be construed as a potential conflict of interest.

## Publisher’s Note

All claims expressed in this article are solely those of the authors and do not necessarily represent those of their affiliated organizations, or those of the publisher, the editors and the reviewers. Any product that may be evaluated in this article, or claim that may be made by its manufacturer, is not guaranteed or endorsed by the publisher.

## Abbreviations

Bic, bicalutamide; ChIP, chromatin immunoprecipitation; CQ, chloroquine; CRPC, castration-resistant prostate cancer; E2, 17β-Estradiol; ERα, estrogen receptor-α; HPLC-MS, high-performance liquid chromatography-mass spectrometry; ERE, estrogen response element; IF, immunofluorescence; IHC, immunohistochemical; Le, letrozole; PCa, prostate cancer; PCSC, prostate cancer stem cell; PRAD, prostate adenocarcinoma; qRT-PCR, quantitative real-time reverse-transcription-polymerase chain reaction; T, Testosterone; TCGA, The Cancer Genome Atlas.
