# Supplementary material for: The HeyL-Aromatase Axis Promotes Cancer Stem Cell Properties by Endogenous Estrogen-Induced Autophagy in Castration-Resistant Prostate Cancer
Source: Front Oncol. 2022 Jan 12;11:787953. doi: 10.3389/fonc.2021.787953 (PMC8789881; doi:10.3389/fonc.2021.787953)
Supplement: Supplementary file 1 [file Presentation_1.pptx]

## Slide 1
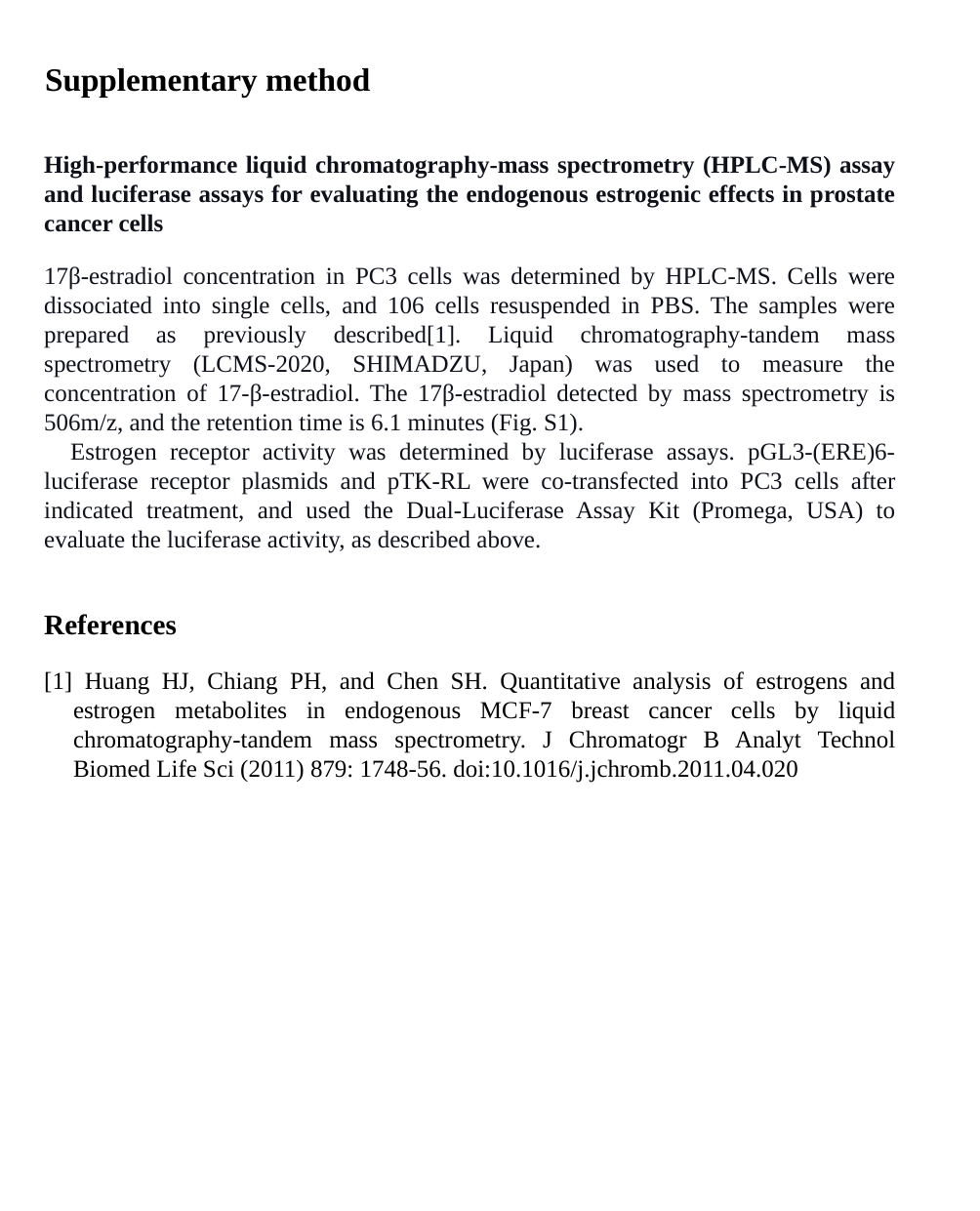

Supplementary method
High-performance liquid chromatography-mass spectrometry (HPLC-MS) assay and luciferase assays for evaluating the endogenous estrogenic effects in prostate cancer cells
17β-estradiol concentration in PC3 cells was determined by HPLC-MS. Cells were dissociated into single cells, and 106 cells resuspended in PBS. The samples were prepared as previously described[1]. Liquid chromatography-tandem mass spectrometry (LCMS-2020, SHIMADZU, Japan) was used to measure the concentration of 17-β-estradiol. The 17β-estradiol detected by mass spectrometry is 506m/z, and the retention time is 6.1 minutes (Fig. S1).
 Estrogen receptor activity was determined by luciferase assays. pGL3-(ERE)6-luciferase receptor plasmids and pTK-RL were co-transfected into PC3 cells after indicated treatment, and used the Dual-Luciferase Assay Kit (Promega, USA) to evaluate the luciferase activity, as described above.
References
[1] Huang HJ, Chiang PH, and Chen SH. Quantitative analysis of estrogens and estrogen metabolites in endogenous MCF-7 breast cancer cells by liquid chromatography-tandem mass spectrometry. J Chromatogr B Analyt Technol Biomed Life Sci (2011) 879: 1748-56. doi:10.1016/j.jchromb.2011.04.020
