# Supplementary material for: The HeyL-Aromatase Axis Promotes Cancer Stem Cell Properties by Endogenous Estrogen-Induced Autophagy in Castration-Resistant Prostate Cancer
Source: Front Oncol. 2022 Jan 12;11:787953. doi: 10.3389/fonc.2021.787953 (PMC8789881; doi:10.3389/fonc.2021.787953)
Supplement: Supplementary file 2 [file Presentation_2.pptx]

## Slide 1
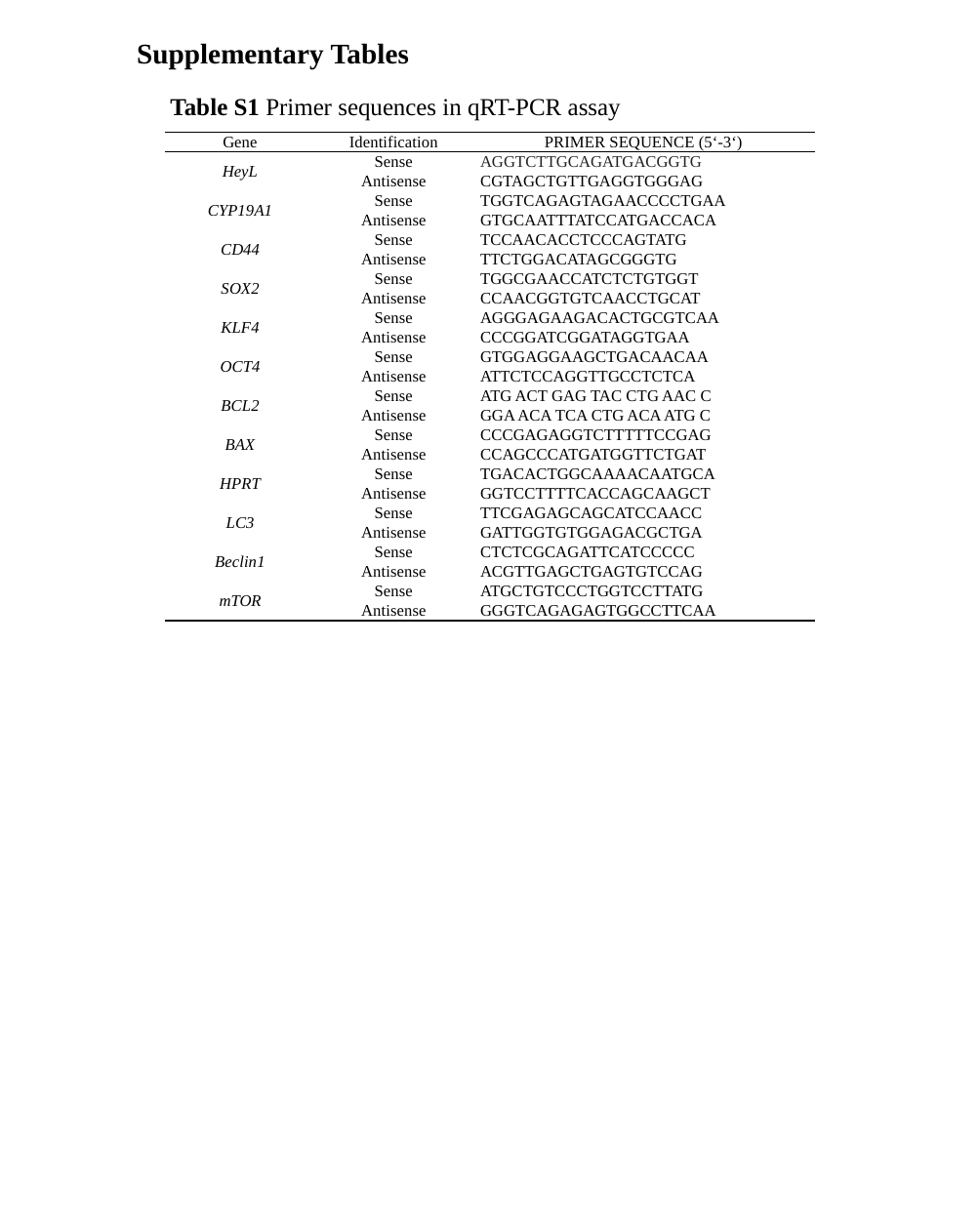

Supplementary Tables
Table S1 Primer sequences in qRT-PCR assay
| Gene | Identification | PRIMER SEQUENCE (5‘-3‘) |
| --- | --- | --- |
| HeyL | Sense | AGGTCTTGCAGATGACGGTG |
| | Antisense | CGTAGCTGTTGAGGTGGGAG |
| CYP19A1 | Sense | TGGTCAGAGTAGAACCCCTGAA |
| | Antisense | GTGCAATTTATCCATGACCACA |
| CD44 | Sense | TCCAACACCTCCCAGTATG |
| | Antisense | TTCTGGACATAGCGGGTG |
| SOX2 | Sense | TGGCGAACCATCTCTGTGGT |
| | Antisense | CCAACGGTGTCAACCTGCAT |
| KLF4 | Sense | AGGGAGAAGACACTGCGTCAA |
| | Antisense | CCCGGATCGGATAGGTGAA |
| OCT4 | Sense | GTGGAGGAAGCTGACAACAA |
| | Antisense | ATTCTCCAGGTTGCCTCTCA |
| BCL2 | Sense | ATG ACT GAG TAC CTG AAC C |
| | Antisense | GGA ACA TCA CTG ACA ATG C |
| BAX | Sense | CCCGAGAGGTCTTTTTCCGAG |
| | Antisense | CCAGCCCATGATGGTTCTGAT |
| HPRT | Sense | TGACACTGGCAAAACAATGCA |
| | Antisense | GGTCCTTTTCACCAGCAAGCT |
| LC3 | Sense | TTCGAGAGCAGCATCCAACC |
| | Antisense | GATTGGTGTGGAGACGCTGA |
| Beclin1 | Sense | CTCTCGCAGATTCATCCCCC |
| | Antisense | ACGTTGAGCTGAGTGTCCAG |
| mTOR | Sense | ATGCTGTCCCTGGTCCTTATG |
| | Antisense | GGGTCAGAGAGTGGCCTTCAA |

## Slide 2
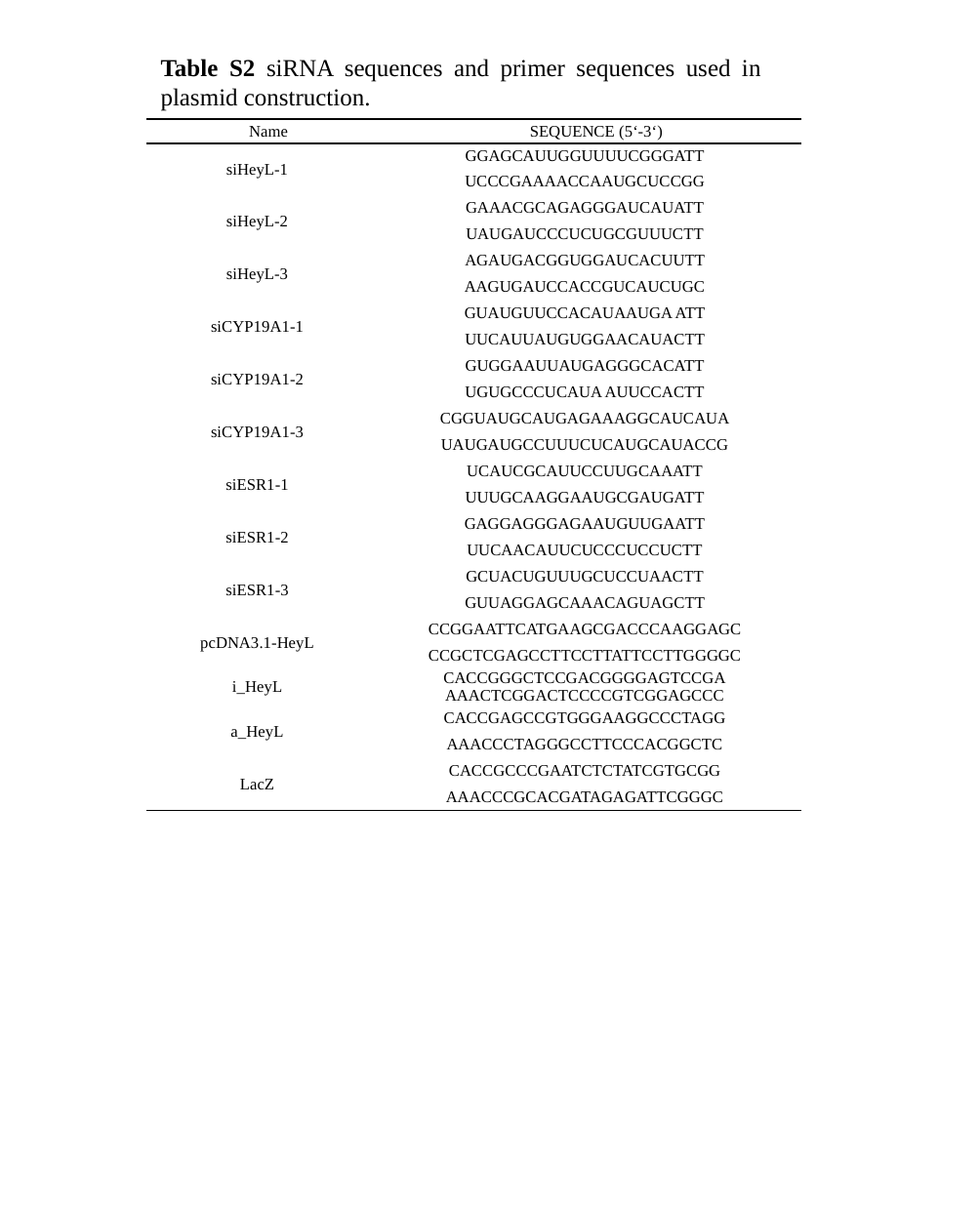

Table S2 siRNA sequences and primer sequences used in plasmid construction.
| Name | | SEQUENCE (5‘-3‘) |
| --- | --- | --- |
| siHeyL-1 | GGAGCAUUGGUUUUCGGGATT | |
| | UCCCGAAAACCAAUGCUCCGG | |
| siHeyL-2 | GAAACGCAGAGGGAUCAUATT | |
| | UAUGAUCCCUCUGCGUUUCTT | |
| siHeyL-3 | AGAUGACGGUGGAUCACUUTT | |
| | AAGUGAUCCACCGUCAUCUGC | |
| siCYP19A1-1 | GUAUGUUCCACAUAAUGA ATT | |
| | UUCAUUAUGUGGAACAUACTT | |
| siCYP19A1-2 | GUGGAAUUAUGAGGGCACATT | |
| | UGUGCCCUCAUA AUUCCACTT | |
| siCYP19A1-3 | CGGUAUGCAUGAGAAAGGCAUCAUA | |
| | UAUGAUGCCUUUCUCAUGCAUACCG | |
| siESR1-1 | UCAUCGCAUUCCUUGCAAATT | |
| | UUUGCAAGGAAUGCGAUGATT | |
| siESR1-2 | GAGGAGGGAGAAUGUUGAATT | |
| | UUCAACAUUCUCCCUCCUCTT | |
| siESR1-3 | GCUACUGUUUGCUCCUAACTT | |
| | GUUAGGAGCAAACAGUAGCTT | |
| pcDNA3.1-HeyL | CCGGAATTCATGAAGCGACCCAAGGAGC | |
| | CCGCTCGAGCCTTCCTTATTCCTTGGGGC | |
| i\_HeyL | CACCGGGCTCCGACGGGGAGTCCGA AAACTCGGACTCCCCGTCGGAGCCC | |
| a\_HeyL | CACCGAGCCGTGGGAAGGCCCTAGG | |
| | AAACCCTAGGGCCTTCCCACGGCTC | |
| LacZ | CACCGCCCGAATCTCTATCGTGCGG | |
| | AAACCCGCACGATAGAGATTCGGGC | |
