# Supplementary material for: The HeyL-Aromatase Axis Promotes Cancer Stem Cell Properties by Endogenous Estrogen-Induced Autophagy in Castration-Resistant Prostate Cancer
Source: Front Oncol. 2022 Jan 12;11:787953. doi: 10.3389/fonc.2021.787953 (PMC8789881; doi:10.3389/fonc.2021.787953)
Supplement: Supplementary file 3 [file Presentation_3.pptx]

## Slide 1
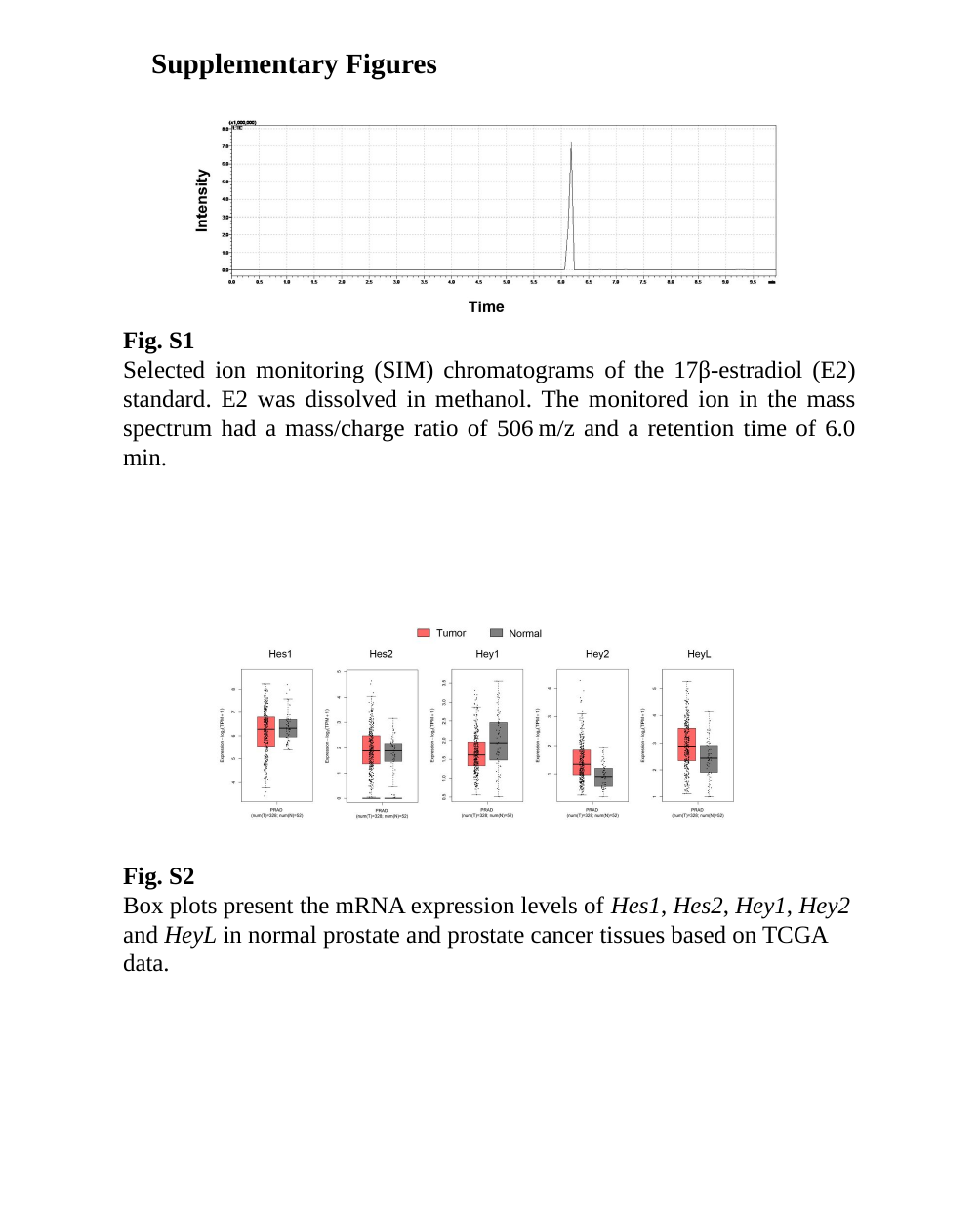

Supplementary Figures
Fig. S1
Selected ion monitoring (SIM) chromatograms of the 17β-estradiol (E2) standard. E2 was dissolved in methanol. The monitored ion in the mass spectrum had a mass/charge ratio of 506 m/z and a retention time of 6.0 min.
Fig. S2
Box plots present the mRNA expression levels of Hes1, Hes2, Hey1, Hey2 and HeyL in normal prostate and prostate cancer tissues based on TCGA data.

## Slide 2
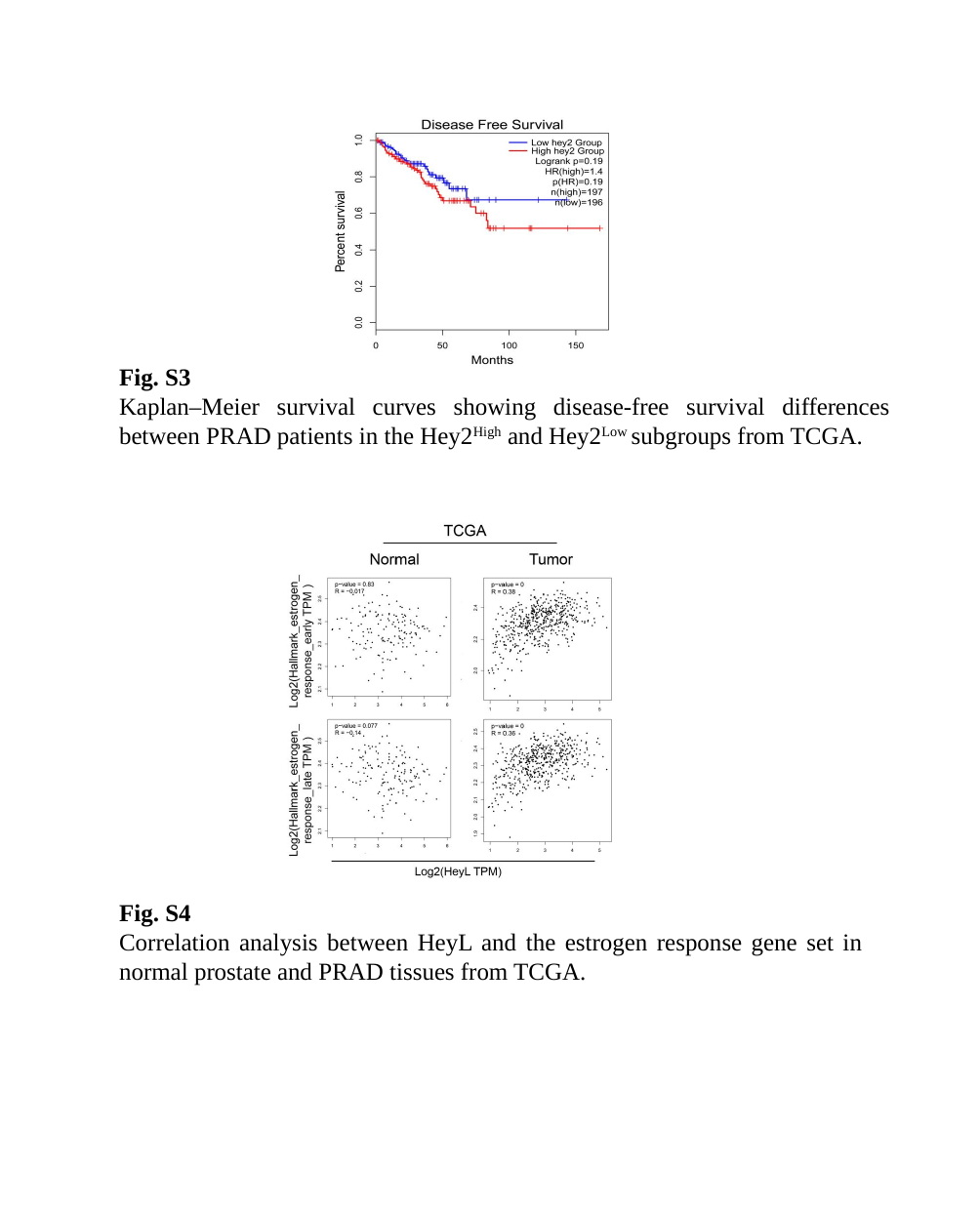

Fig. S3
Kaplan–Meier survival curves showing disease-free survival differences between PRAD patients in the Hey2High and Hey2Low subgroups from TCGA.
Fig. S4
Correlation analysis between HeyL and the estrogen response gene set in normal prostate and PRAD tissues from TCGA.

## Slide 3
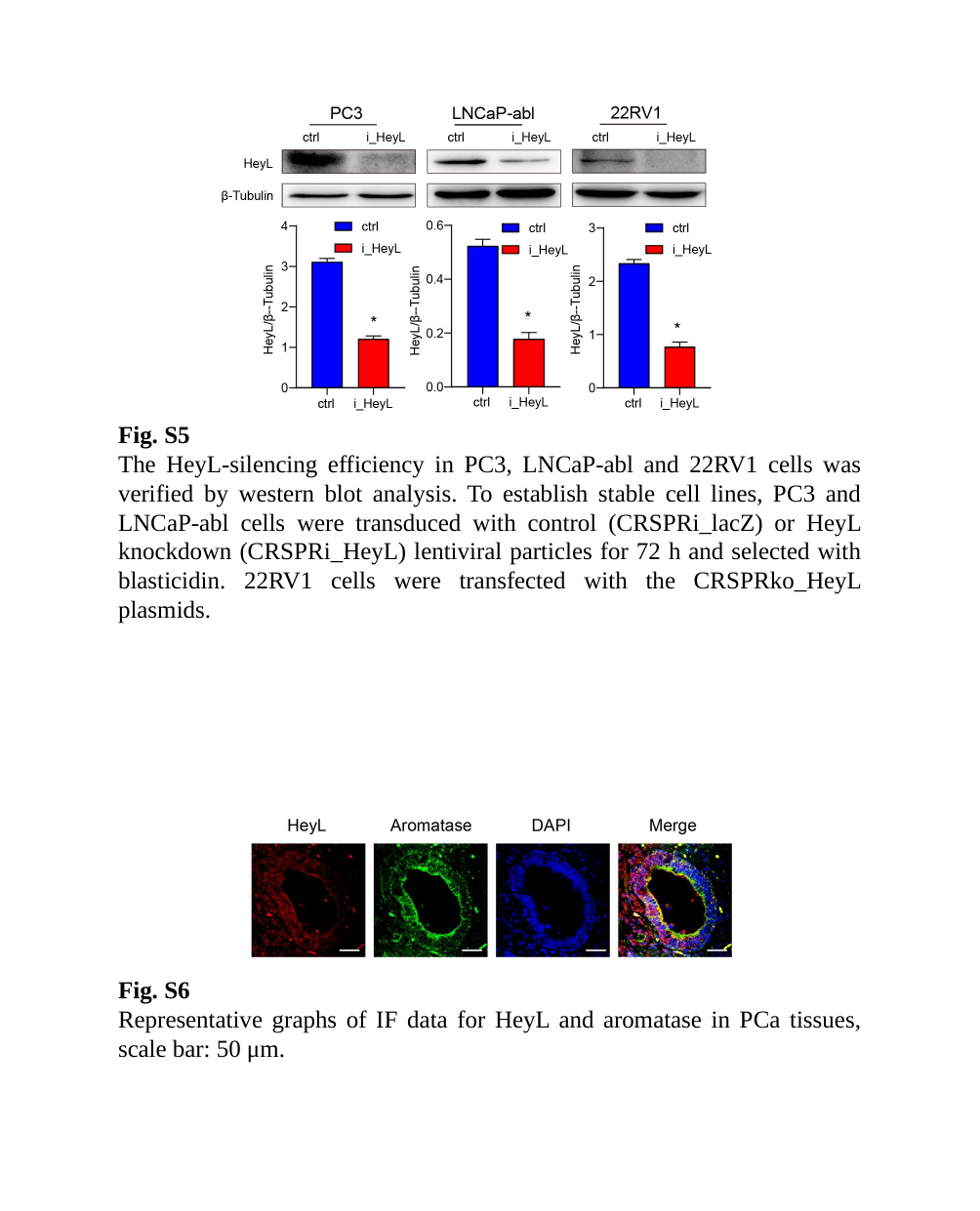

Fig. S5
The HeyL-silencing efficiency in PC3, LNCaP-abl and 22RV1 cells was verified by western blot analysis. To establish stable cell lines, PC3 and LNCaP-abl cells were transduced with control (CRSPRi_lacZ) or HeyL knockdown (CRSPRi_HeyL) lentiviral particles for 72 h and selected with blasticidin. 22RV1 cells were transfected with the CRSPRko_HeyL plasmids.
Fig. S6
Representative graphs of IF data for HeyL and aromatase in PCa tissues, scale bar: 50 μm.

## Slide 4
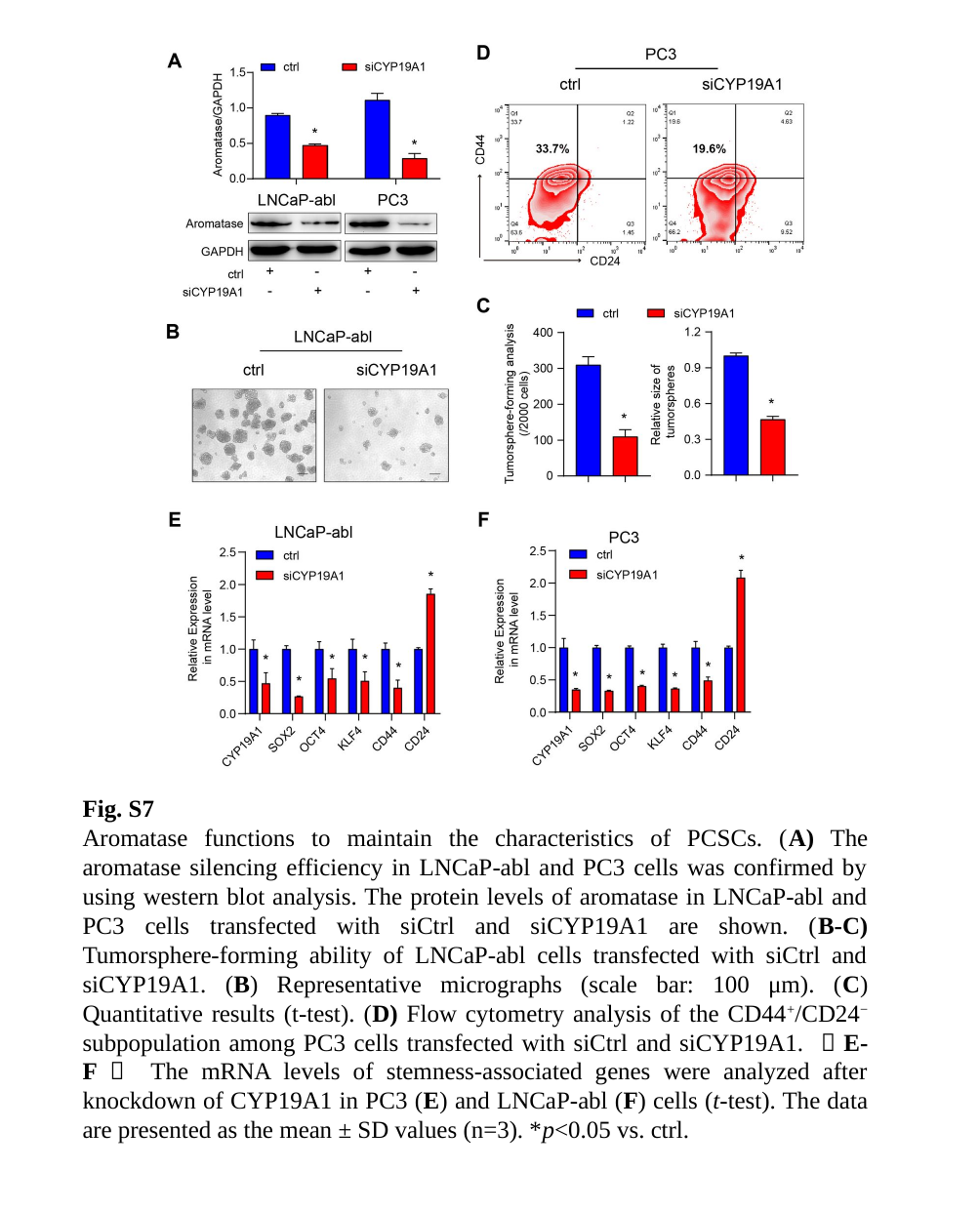

Fig. S7
Aromatase functions to maintain the characteristics of PCSCs. (A) The aromatase silencing efficiency in LNCaP-abl and PC3 cells was confirmed by using western blot analysis. The protein levels of aromatase in LNCaP-abl and PC3 cells transfected with siCtrl and siCYP19A1 are shown. (B-C) Tumorsphere-forming ability of LNCaP-abl cells transfected with siCtrl and siCYP19A1. (B) Representative micrographs (scale bar: 100 μm). (C) Quantitative results (t-test). (D) Flow cytometry analysis of the CD44+/CD24− subpopulation among PC3 cells transfected with siCtrl and siCYP19A1. （E-F） The mRNA levels of stemness-associated genes were analyzed after knockdown of CYP19A1 in PC3 (E) and LNCaP-abl (F) cells (t‐test). The data are presented as the mean ± SD values (n=3). *p<0.05 vs. ctrl.

## Slide 5
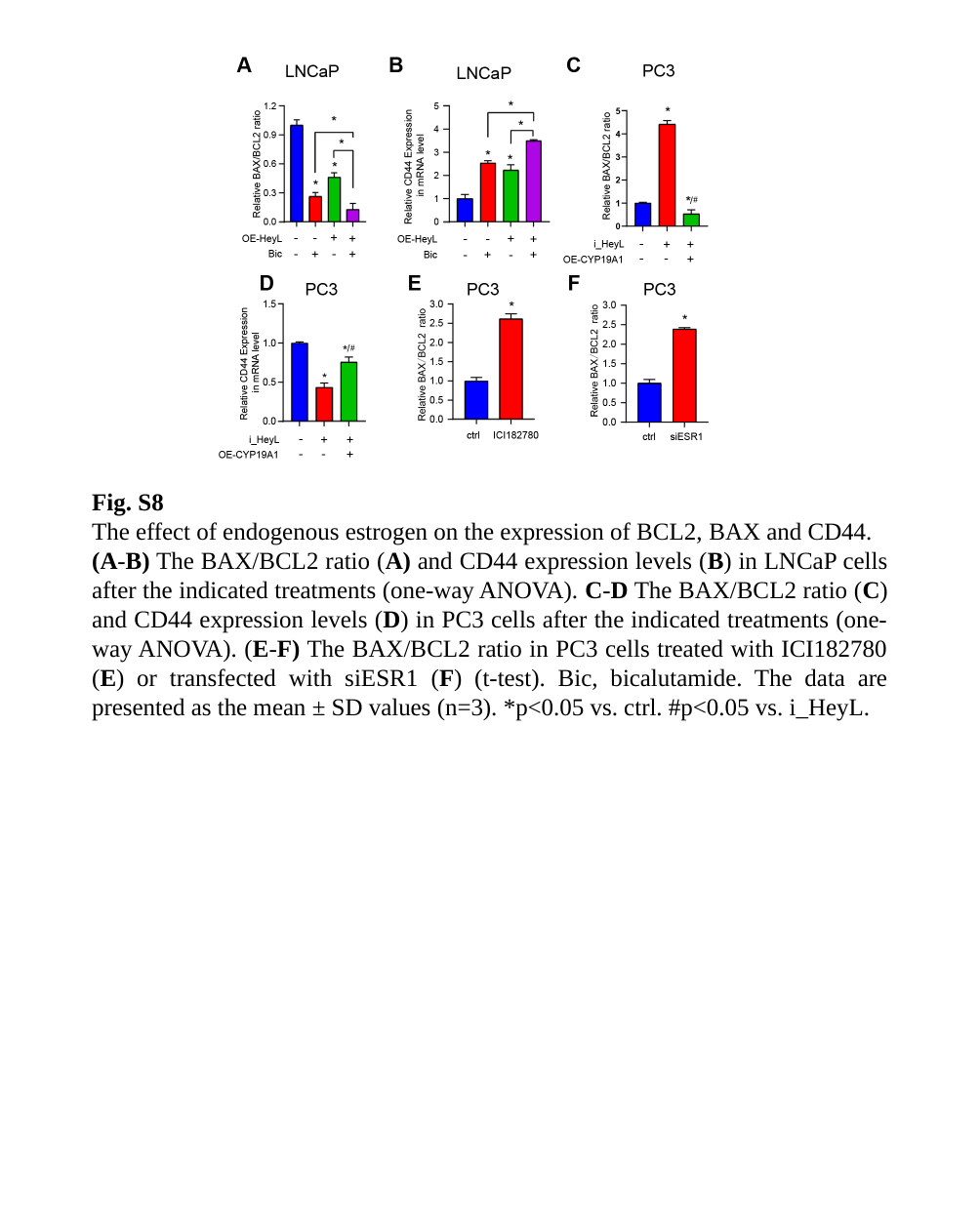

Fig. S8
The effect of endogenous estrogen on the expression of BCL2, BAX and CD44.
(A-B) The BAX/BCL2 ratio (A) and CD44 expression levels (B) in LNCaP cells after the indicated treatments (one-way ANOVA). C-D The BAX/BCL2 ratio (C) and CD44 expression levels (D) in PC3 cells after the indicated treatments (one-way ANOVA). (E-F) The BAX/BCL2 ratio in PC3 cells treated with ICI182780 (E) or transfected with siESR1 (F) (t-test). Bic, bicalutamide. The data are presented as the mean ± SD values (n=3). *p<0.05 vs. ctrl. #p<0.05 vs. i_HeyL.

## Slide 6
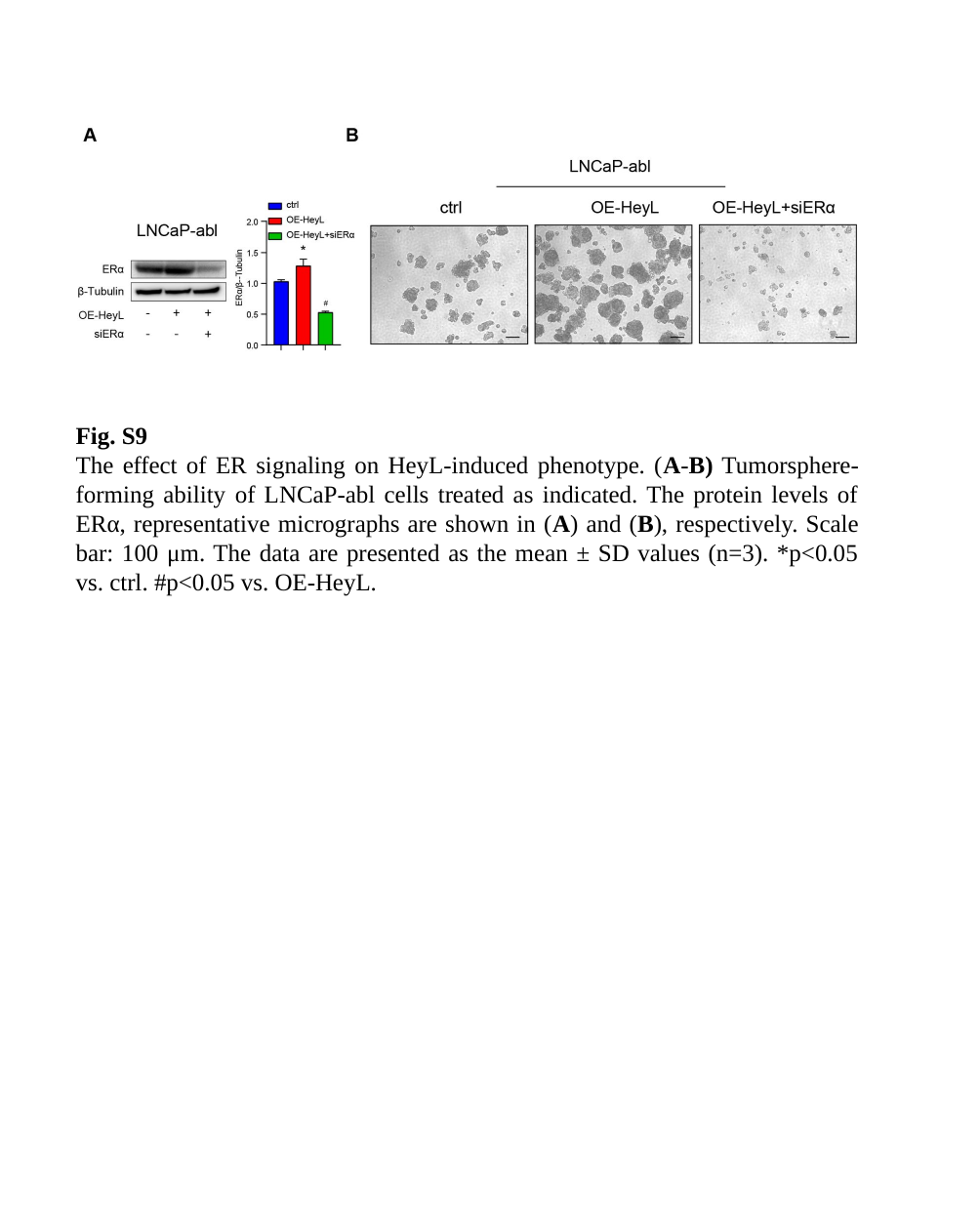

Fig. S9
The effect of ER signaling on HeyL-induced phenotype. (A-B) Tumorsphere-forming ability of LNCaP-abl cells treated as indicated. The protein levels of ERα, representative micrographs are shown in (A) and (B), respectively. Scale bar: 100 μm. The data are presented as the mean ± SD values (n=3). *p<0.05 vs. ctrl. #p<0.05 vs. OE-HeyL.

## Slide 7
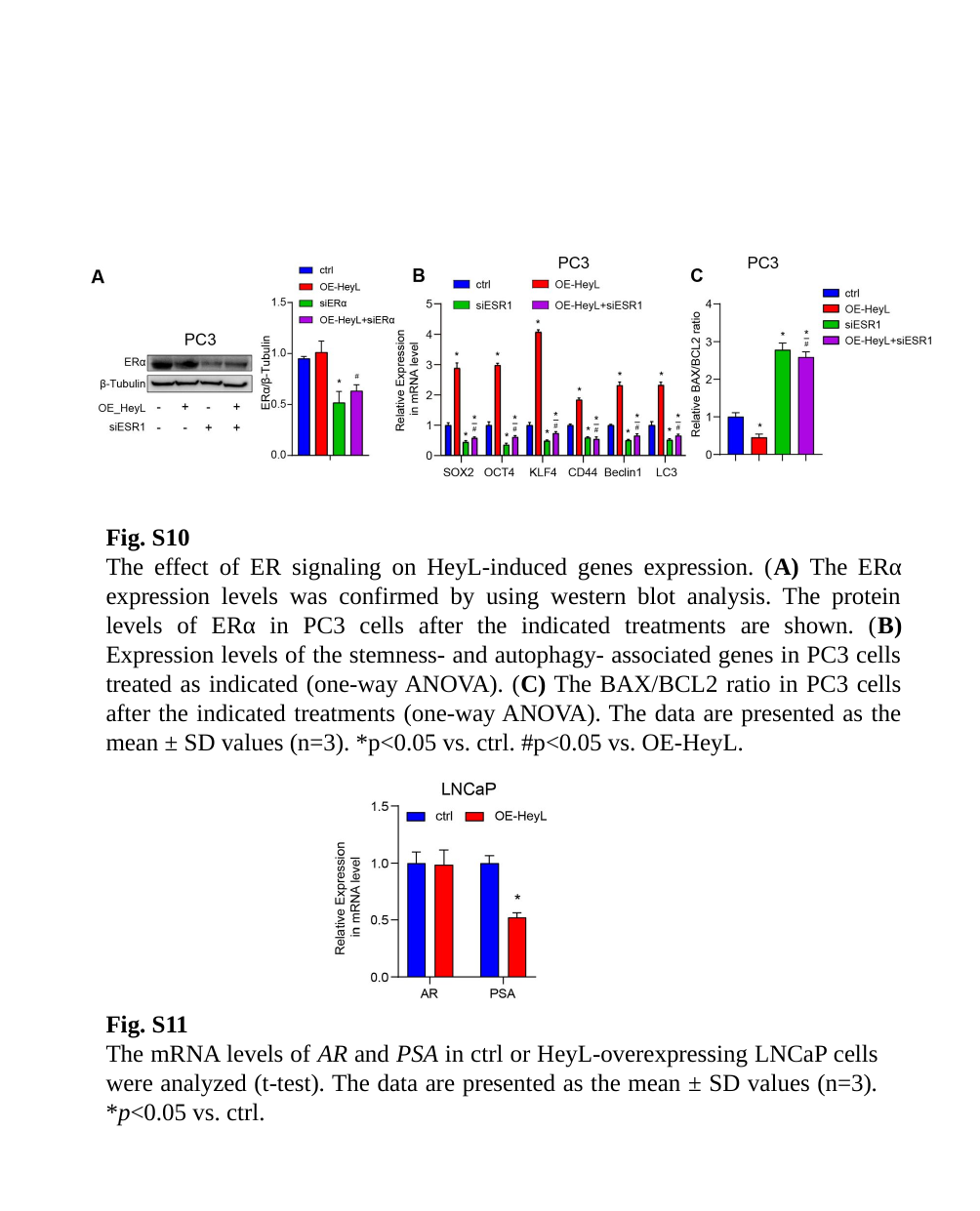

Fig. S10
The effect of ER signaling on HeyL-induced genes expression. (A) The ERα expression levels was confirmed by using western blot analysis. The protein levels of ERα in PC3 cells after the indicated treatments are shown. (B) Expression levels of the stemness- and autophagy- associated genes in PC3 cells treated as indicated (one-way ANOVA). (C) The BAX/BCL2 ratio in PC3 cells after the indicated treatments (one-way ANOVA). The data are presented as the mean ± SD values (n=3). *p<0.05 vs. ctrl. #p<0.05 vs. OE-HeyL.
Fig. S11
The mRNA levels of AR and PSA in ctrl or HeyL-overexpressing LNCaP cells were analyzed (t-test). The data are presented as the mean ± SD values (n=3). *p<0.05 vs. ctrl.
